# Neuropsychiatric symptom subtypes associated with Alzheimer's disease and related dementia neuropathologic changes in the National Alzheimer's Coordinating Center neuropathology cohort

**DOI:** 10.1002/alz70857_106793

**Published:** 2025-12-25

**Authors:** Brenna Cholerton, Gary W Beecham, Christiane Reitz, Alyssa N. De Vito, Michael L Cuccaro, Walter W. Kukull, Thomas J. Montine, Edward D. Huey

**Affiliations:** ^1^ Department of Pathology, Stanford University, Stanford, CA, USA; ^2^ Wake Forest University School of Medicine, Winston‐Salem, NC, USA; ^3^ Columbia University Irving Medical Center, New York, NY, USA; ^4^ Brown University Medical School, Providence, RI, USA; ^5^ Dr. John T. Macdonald Foundation Department of Human Genetics, University of Miami Miller School of Medicine, Miami, FL, USA; ^6^ Department of Medicine, University of Washington School of Medicine, Seattle, WA, USA; ^7^ Department of Pathology, Stanford University School of Medicine, Stanford, CA, USA; ^8^ Department of Psychiatry and Human Behavior, Alpert Medical School of Brown University, Providence, RI, USA

## Abstract

**Background:**

Neuropsychiatric symptoms (NPS) are highly prevalent in Alzheimer's disease (AD) and related dementias and are responsible for a substantial proportion of disease‐associated burden. Differences in timing and type of NPSmay result from distinct underlying diseases and may be important in predicting outcomes. As such, we examined whether specific NPS clusters (early psychosis/agitation [EPA], late psychosis/agitation [LPA], or affective symptoms [AS]) had distinct associations with common dementia‐associated neuropathologic lesions and/or total neuropathologic burden. Additionally, we assessed whether AD neuropathologic change (ADNC) in the presence or absence of non‐ADNC lesions had differential associations with NPS subtypes.

**Method:**

Data were obtained from the National Alzheimer's Coordinating Center (NACC) Uniform and Neuropathology datasets. NPS subtypes were retroactively assigned using the Neuropsychiatric Inventory Questionnaire. Neuropathologic lesions were described in harmonized form across NACC sites. Adjusted logistic regression analyses estimated associations between NPS subtypes and individual neuropathologic changes, total neuropathologic burden, and ADNC in the presence/absence of non‐ADNCs.

**Result:**

Among participants diagnosed with dementia (*n* = 2352), EPA was associated with Lewy body (OR=2.4, *p* <0.0001) and white matter (OR=1.7, *p* <0.001) pathology. LPA was associated with neurofibrillary tangles (OR=2.6, *p* <0.0001), neuritic plaques (OR=2.1, *p* <0.0001), Lewy bodies (OR=1.9, *p* <0.0001), and cerebral amyloid angiopathy (OR=1.6, *p* <0.0001). AS was associated with white matter disease (OR=2.8, *p* <0.0001) and neurofibrillary tangles (OR=1.7, *p* <0.001). EPA was associated with increased odds of LBD/no ADNC (OR=3.6, *p* <0.001), while LPA was associated with increased odds of ADNC/no LBD (OR=3.0, *p* <0.0001). LPA alone was associated with ADNC/no WMD (OR=3.3, *p* <0.0001), while both LPA and EPA were all associated with ADNC+WMD (ORs=2.9, 3.6, p values<0.0001). All subtypes were associated with increased total neuropathologic burden.

**Conclusion:**

We found that timing of symptom onset and specific symptom cluster had distinct patterns of association with neuropathologic lesions, and that these may differ according to the presence and severity of concurrent ADNC. Additionally, we report that NPS increased significantly with the number of neuropathologic changes present, providing support for reducing total neuropathologic burden, perhaps through lifestyle modification and disease‐modifying treatments, as a method for harm‐reduction in dementia.

